# Clinical efficacy of Yinhua Miyanling tablet in the treatment of uncomplicated urinary tract infections: A systematic review and meta-analysis

**DOI:** 10.1097/MD.0000000000045299

**Published:** 2025-10-31

**Authors:** Jiasen Ding, Yanyan Zhou, Ming Li, Yiwen Tang, Xiong Wang, Qi Li, Ding Li, Feng Xu, Ruoyu Qiao, Zejia Zhang, Yongqiang Wei, Pengyu Cheng, Lihong Liu, Rongfu Ji, Xiaofeng Jiang, Zhan Gao

**Affiliations:** a Department of Urology, Xiyuan Hospital, China Academy of Chinese Medical Sciences, Beijing, China; b Department of Graduate School, China Academy of Chinese Medical Sciences, Beijing, China; c Department of Graduate School, Beijing University of Chinese Medicine, Beijing, China.

**Keywords:** GRADE, meta-analysis, randomized controlled trial, uncomplicated urinary tract infections, UTIs, Yinhua Miyanling tablet

## Abstract

**Background:**

Although Yinhua Miyanling tablet has demonstrated promising efficacy in managing uncomplicated urinary tract infections (UTIs), robust clinical evidence from large-scale studies remains scarce. This systematic review aims to comprehensively evaluate the therapeutic effectiveness and safety profile of Yinhua Miyanling tablet in the treatment of uncomplicated UTIs, providing an evidence-based assessment for clinical practice.

**Methods:**

A comprehensive computerized search was conducted across multiple databases, including China National Knowledge Infrastructure, China Science and Technology Journal Database, Wanfang Data, Chinese Biomedical Literature Database, Big Medical Knowledge Base, Medline, PubMed, Embase, Cochrane Library, and Web of Science. The search period spanned from the inception of each database to October 2023, aiming to identify randomized controlled trials (RCTs) investigating the efficacy of Yinhua Miyanling tablets in treating uncomplicated UTIs. The selected studies were subjected to meta-analysis using RevMan 5.3 software (The Cochrane Collaboration, London, United Kingdom). The methodological quality of the included literature was assessed using the Cochrane Risk of Bias Tool (version 5.1.0; The Cochrane Collaboration, London, United Kingdom) and the modified Jadad scale. Additionally, the GRADE system was employed to evaluate the overall quality of evidence.

**Results:**

A total of 14 RCTs, involving 1773 patients, were included in the meta-analysis. The results demonstrated that Yinhua Miyanling tablets, whether used alone or in combination with Western medicine, significantly improved clinical efficacy and bacterial clearance rates while reducing recurrence rates (all *P* < .05). Notably, there was no statistically significant difference in adverse reactions between the treatment and control groups, suggesting a favorable safety profile.

**Conclusion:**

The Yinhua Miyanling tablet demonstrates therapeutic potential in managing uncomplicated UTIs. However, the current evidence is limited by the relatively low methodological quality of included studies. Further validation through large-scale, multicenter RCTs with rigorous designs is warranted to strengthen these findings. According to the Grading of Recommendations Assessment, Development and Evaluation evidence assessment, the available data provide moderate confidence in the efficacy of Yinhua Miyanling tablet for treating uncomplicated UTIs.

## 1. Introduction

Urinary tract infection (UTI) refers to microbial infections affecting any part of the urinary system, including the kidneys (pyelonephritis), ureters, bladder (cystitis), and urethra (urethritis).^[1]^

Based on urinary tract status at the time of infection, UTIs can be classified into 2 categories: uncomplicated and complicated. Uncomplicated UTIs occur in individuals with structurally and functionally normal urinary tracts and without underlying comorbidities such as diabetes mellitus or immunocompromised states.^[2]^

In clinical practice, uncomplicated UTIs are typically treated with antibiotics. However, prolonged and repeated antibiotic use has led to increased bacterial resistance and higher recurrence rates. Modern research demonstrates that traditional Chinese medicine (TCM) offers distinct therapeutic advantages, including multi-target effects and favorable safety profiles. TCM interventions have shown promising results in UTI management by potentially reducing antibiotic requirements, shortening treatment duration, enhancing therapeutic efficacy, and decreasing recurrence rates.^[3,4]^

Yinhua Miyanling tablet is made from the traditional Chinese medicine prescription Bazheng San. It has the effects of clearing heat, detoxifying, and dispelling dampness. It is a pure Chinese medicine preparation composed of honeysuckle flower, Banzhi lian, Shiwei, Qu Mai, polygonum, Semen Plantaginis, Herba Lophatheri; clematis caulis, Herba Taxilli, and rushes.^[5]^ Emerging research indicates that Yinhua Miyanling tablets exhibit broad-spectrum antimicrobial activity with favorable safety profiles. Notably, these tablets demonstrate a low propensity for inducing bacterial resistance even with prolonged administration, suggesting potential advantages over conventional antibiotic therapies.^[6]^ Yinhua Miyanling tablet is a widely used clinical treatment for uncomplicated UTIs. While numerous clinical studies have investigated its efficacy, no comprehensive systematic review has been published to date. This study conducted a systematic evaluation of Yinhua Miyanling tablet’s therapeutic effectiveness and safety profile in treating uncomplicated UTIs. Our findings provide robust evidence-based medical support for its clinical application, offering valuable guidance for therapeutic decision-making.

## 2. Data and methods

### 2.1. Inclusion and exclusion criteria

#### 2.1.1. Study type

Randomized controlled trial (RCT) of Yinhua Miyanling tablet in the treatment of uncomplicated UTIs, blinded or not, limited to English and Chinese.

#### 2.1.2. Subjects

Patients with uncomplicated UTIs were diagnosed according to the criteria of the Chinese Guidelines for The Diagnosis and Treatment of Urological Diseases (2022 edition). The patient’s gender, region, nationality, occupation, and course of disease are not limited.

#### 2.1.3. Intervention measures

The experimental group received either the same treatment as the control group or a combination therapy, while the control group was treated with either Western medicine or proprietary Chinese medicine alone. No restrictions were imposed on the total daily dosage or duration of treatment.

#### 2.1.4. Outcome indicators

Clinical effective rate; bacterial clearance rate; recurrence rate; adverse drug reactions; symptom improvement time. According to different evaluation criteria, the total clinical effective rate was divided into 2 categories. One was divided into 4 categories: cure, obvious effect, effective, and ineffective. The other is to divide the curative effect into 3 cases: cure, improvement, and ineffective. Total effective rate = cure rate + effective rate.

#### 2.1.5. Exclusion criteria

Test subjects were only targeted at specific groups such as women, children, and the elderly; repeated publication of literature; the loss of follow-up rate of the subjects was >20%. Studies where full text was not available or where corresponding outcome measures were not provided. Nonclinical trial studies, such as reviews, famous medical records, case reports, animal experiments, etc. Only one treatment regimen was used, with no control group.

### 2.2. Retrieval strategy

Computer search CNKI, China Science and Technology Journal Database, Wanfang Data Knowledge Service platform, Chinese Biomedical Literature Database platform, Medical Knowledge Base, Medline, PubMed, Embase, Cochrane Library, Web of Science, and other databases were used to collect the RCT of Yinhua Miyanling tablet in the treatment of uncomplicated UTIs, and the search time was from the establishment of the database to October 2023. In addition, references to the included literature were traced to supplement access to relevant literature. Chinese search terms included: “Yinhua Miyanling tablet,” “urinary tract infection,” and “urinary tract infection.” English search terms are searched according to different database features by combining MeSH or Emtree subject words with free words. The search terms “Yinhua miyanling tablet” and “urinary tract infection” are supplemented by manual search. The search terms were “Yinhua Miyanling tablet” and “acute cystitis” OR “acute pyelonephritis.” Taking CNKI as an example, the search strategy was “SU= Yinhua miyanling Tablet “*” Urinary tract infection “+” Yinhua minyanling tablet “*” urinary tract infection”.

### 2.3. Literature screening and data extraction

The 2 researchers independently screened the literature according to the formulated exclusion criteria, filled in and summarized the relevant data, and discussed and negotiated with the third researcher in case of differences. When the included literature is not fully available, contact the original author to obtain as complete information as possible. The extracted information included: general data (such as author, year of publication, journal, age range of cases, course of disease, course of treatment, and sample size), randomization method, assignment hiding, blind method, intervention measures of trial group, and control group, outcome indicators (effective rate, bacterial clearance rate, recurrence rate, and adverse drug reactions), follow-up data, etc.

### 2.4. Bias risk assessment for included studies

The included literature was evaluated independently by 2 investigators using the “Bias risk assessment” tool in the Cochrane Handbook 5.1.0 (The Cochrane Collaboration, London, United Kingdom), and if the bias risk assessment was inconsistent, it was resolved by discussion and negotiation with the third investigator. The bias assessment mainly involves 6 aspects and 7 items, including the generation of random sequences (selection bias), assignment concealment (selection bias), blinding of investigators and subjects (implementation bias), blind evaluation of study outcomes (measurement bias), integrity of outcome data (follow-up bias), selective reporting of study results (reporting bias), and other sources (other bias).

### 2.5. Statistical analysis

Meta-analysis of relevant data was performed using RevMan5.3 software (The Cochrane Collaboration). The odds ratio (OR) was selected for the bivariate effect index, and a 95% confidence interval (CI) was used for interval estimation. *I*^2^ was used to test the heterogeneity of the included RCT studies. If the results were homogeneous (*P* ≥ .10, *I*^2^ ≤ 50%), the fixed-effect model was used for meta-analysis. On the contrary, when there is statistical heterogeneity, the first choice is to check whether the original data is incorrect. If the original data is correct, subgroup analysis, meta-regression, or sensitivity analysis can be performed; if the cause still cannot be explained, the random effects model is used for meta-analysis. *P* < .05 indicated that the difference was statistically significant.

### 2.6. Evidence quality rating

We used GRADEpro (version 3.5; Evidence Prime Inc, Hamilton, Ontario, Canada) to rate the quality of evidence of the incidences using the Grading of Recommendations Assessment, Development and Evaluation (GRADE) system. Multiple researchers participated in the process. The quality of the evidence was divided into 4 categories: “high,” “moderate,” “low,” and “very low.” Randomized trials were considered as high-quality evidence, and observational studies were considered as low-quality evidence. Five factors that can reduce the quality of evidence included limitations, inconsistency, indirectness, imprecision, and publication bias. Three factors that can increase the quality of evidence consisted of large effect, plausible residual confounding, and dose–response gradient.

## 3. Results

### 3.1. Literature screening process and results

**Figure 1.**
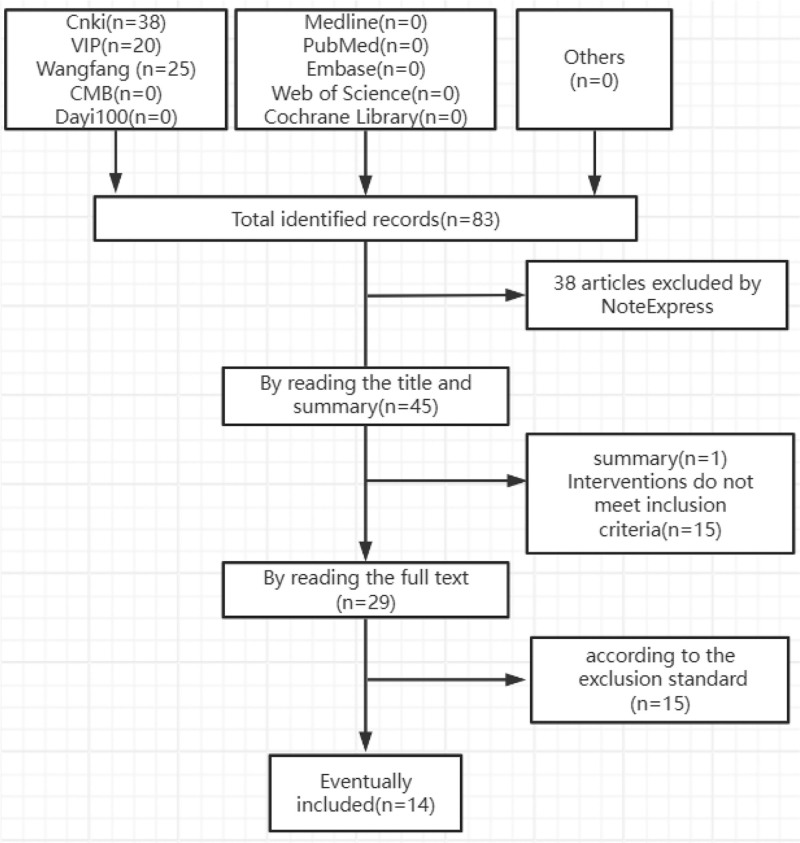
Literature screening process.

Initial database searches identified 83 potentially relevant articles, all published in Chinese. After removing 38 duplicate records using NoteExpress software (Beijing Aegean Sea Lezhi Technology Co., Ltd., Beijing, China), we screened the remaining 45 articles by title and abstract, excluding 16 that did not meet our inclusion criteria. Full-text review of the remaining 29 articles led to the exclusion of 15 non-randomized studies. Ultimately, 14 RCTs involving a total of 1773 participants were included in our analysis.^[7–20]^ Figure 1 presents the complete study selection process in PRISMA flow diagram format.

### 3.2. Basic characteristics of included studies and results of bias risk assessment

**Figure 2.**
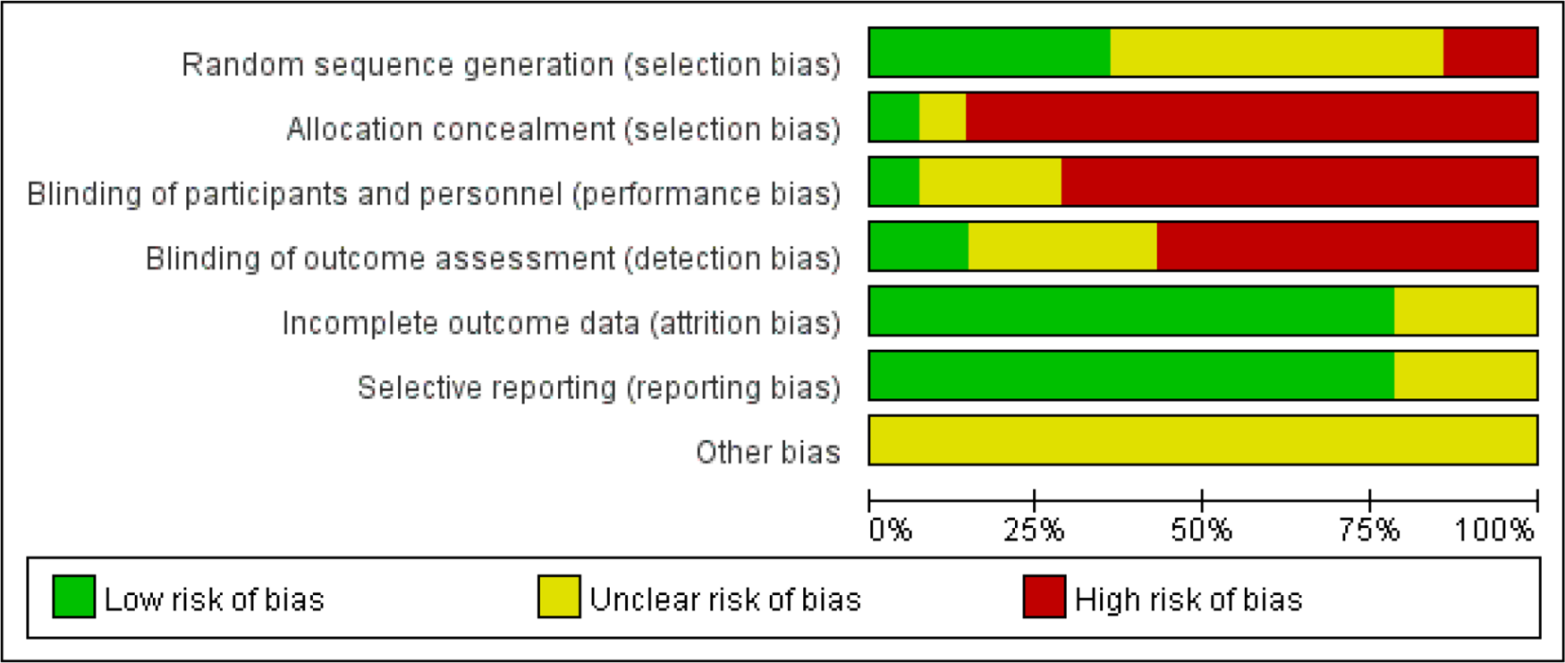
Bias risk graph.

**Figure 3.**
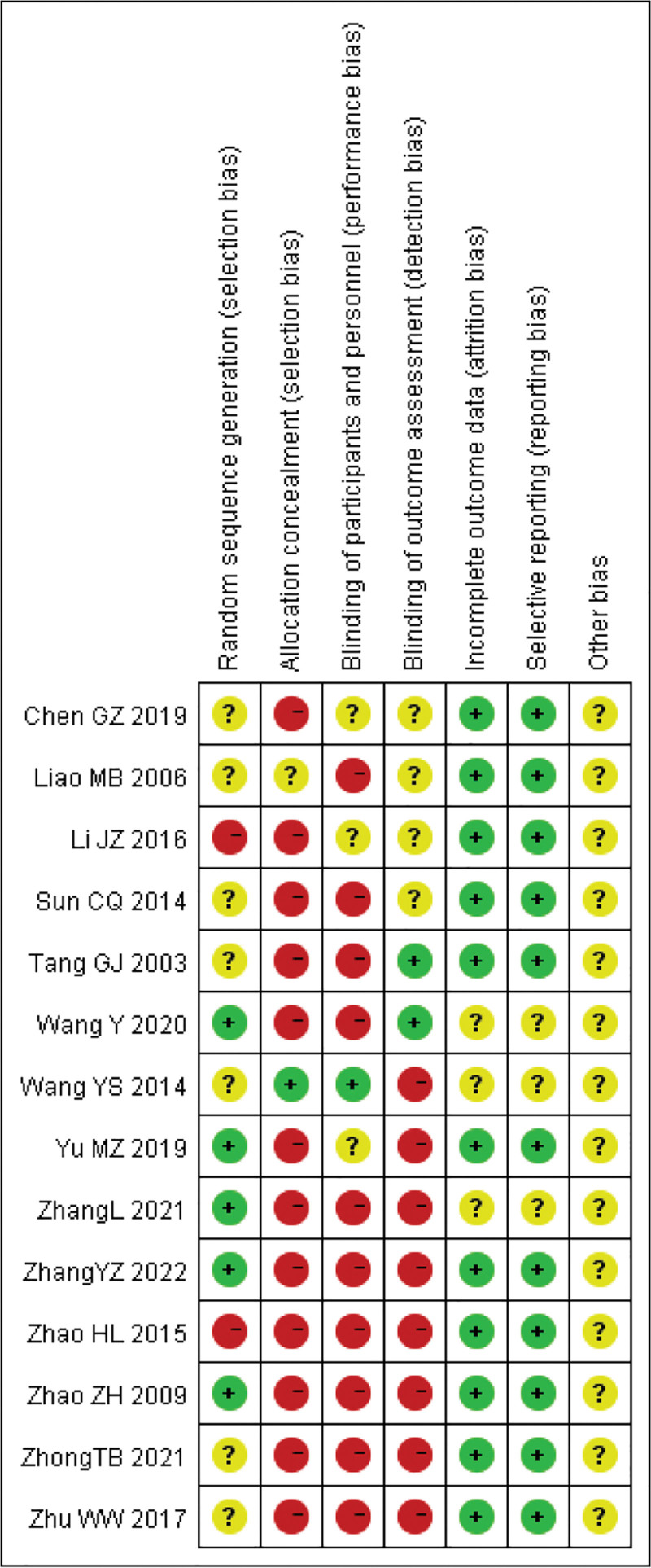
Summary of risk of bias by study.

YT: Yinhua Miyanling tablet; LC: Levofloxacin; CC: Ciprofloxacin; AT: Aztreonam; ST: Sanjin tablet; NN: Niaoganning; QG: Qingling granule; effective rate; recurrence rate; bacterial clearance; adverse reaction; symptom improvement time.

The basic characteristics of the included studies are shown in Table 1. The intervention measures of the experimental group were Yinhua Miyanling tablet alone or combined with a levofloxacin capsule, and the intervention measures of the control group were positive control drugs. All baseline levels were described, with no statistical difference and no comparability. Out of the 14 studies, only 6 studies^[10,11,14,17–19]^ mentioned randomized methods, and the rest did not, all studies did not mention blinding and hiding measures, and 5 studies^[8,10–13]^ had relevant follow-up. The evaluation was conducted according to the “Bias risk assessment” tool in the Cochrane Handbook 5.1.0. The risk assessment of bias for included studies is shown in Figure 2, and the specific risk summary of bias for each study is shown in Figure 3.

**Table 1:** Basic features of included studies.

References	Group	Number of cases	Age (yr)	The course of illness (d)	Intervening measure	The course of treatment (d)	Outcome indicator
Zhao^[7]^	Experimental group	28	18–70	–	YT + ST	14	①
	Control group	26	17–70	–	ST	14	
Zhu et al^[8]^	Experimental group	47	33.4 ± 5.6	3.4 ± 1.5	YT + LC	7–14	①②③④
	Control group	46	32.5 ± 5.7	3.2 ± 1.6	LC	7–14	
Sun and Li^[9]^	Experimental group	35	34 ± 2.13	–	YT + LC	14	①④
	Control group	35	33 ± 2.01	–	LC	14	
Yu et al^[10]^	Experimental group	90	44.9 ± 8.1	4.5 ± 1.2	YT + LC	14	①④
	Control group	90	45.4 ± 6.8	4.5 ± 1.5	LC	14	
Zhao and Gao^[11]^	Experimental group	35	43 ± 3.5	–	YT + LC	7	①④
	Control group	35	43 ± 3.5	–	NN	7	
Liao et al^[12]^	Experimental group	420	18–65	–	YT	14	①④
	Control group	110	18–65	–	QG	14	
Wang and Wang^[13]^	Experimental group	40	31.2 ± 5.4	6.9 ± 1.9	YT + LC	7–14	①②③
	Control group	40	30.3 ± 5.1	7.0 ± 1.5	LC	7–14	
Li^[14]^	Experimental group	60	43 ± 11.3	–	YT	14	①
	Control group	60	43 ± 12.6	–	ST	14	
Tang et al^[15]^	Experimental group	40	18–65	–	YT	14	①
	Control group	40	18–65	–	CC	14	
Chen and Zhang^[16]^	Experimental group	75	44.56 ± 4.67	5.42 ± 1.69	YT + LC	14	①④
	Control group	75	44.38 ± 4.57	5.39 ± 1.74	LC	14	
Wang^[17]^	Experimental group	35	36.12 ± 5.12	–	YT	42	①④⑤
	Control group	35	37.48 ± 5.16	–	LC	42	
Zhang and Liu^[18]^	Experimental group	35	44.43 ± 3.64	–	YT + LC	14	①
	Control group	35	44.59 ± 3.87	–	LC	14	
Zhang^[19]^	Experimental group	53	36.8 ± 5.9	3.5 ± 0.76	YT + AT	10	①④
	Control group	53	37.7 ± 6.1	3.9 ± 0.8	AT	10	
Zhong^[20]^	Experimental group	50	40.45 ± 5.12	–	YT + LC	30	①
	Control group	50	41.89 ± 1.02	–	LC	30	

All the outcome measures (①②③④) were significant in the corresponding articles.

Among the 14 RCTs analyzed, there have 5 studies (35.7%) reported adequate sequence generation using different randomization. The remaining studies merely mentioned “randomization” without specifying methods (Fig. 3). Allocation concealment was properly implemented in only 1 trials (7.1%) through sealed opaque envelopes.

Notably, 100% (14/14) of the included studies failed to describe blinding procedures for outcome assessors, introducing potential detection bias.

### 3.3. Results of meta-analysis

#### 3.3.1. Clinical response rate

**Figure 4.**
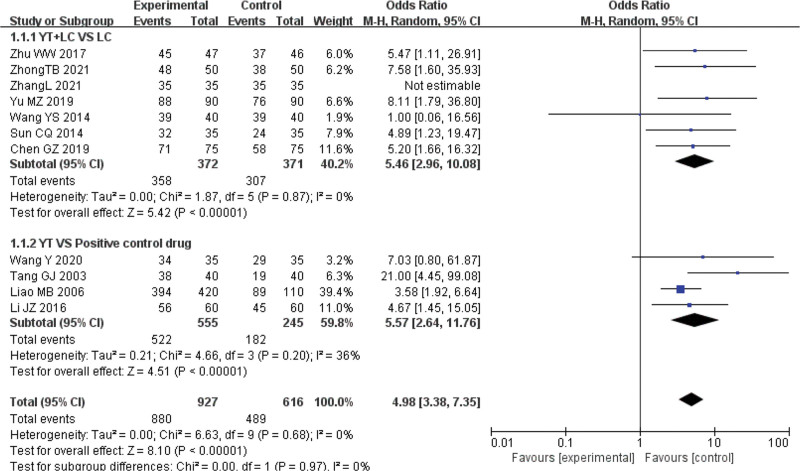
Clinical response rate: Yinhua Miyanling tablet + Levofloxacin capsules versus Levofloxacin capsules Yinhua Miyanling tablet versus positive control drug.

Seven studies^[8–10,13,16,18,20]^ were conducted to compare the effect of Yinhua Miyanling tablet + Levofloxacin capsule vs Levofloxacin capsule on clinical response rate, with 372 patients in the experimental group and 371 patients in the control group. Heterogeneity test results showed that *P* = .87; and *I*^2^ = 0%, clinical homogeneity, fixed effect model, OR = 5.42, 95% CI (2.96, 10.08), *P* ˂ .01. Four studies^[12,14,15,17]^ were conducted to compare the effect of Yinhua Miyanling tablet versus positive control on clinical response rate, with 555 patients in the experimental group and 245 patients in the control group. Heterogeneity test results showed that *P* = .20; *I*^2^ = 36%, Using the fixed-effect model, the results showed that OR = 4.51, 95% CI was (2.64–11.76), *P* ˂ .01, the clinical effective rate was statistically significant. See Figure 4. While pooled analysis showed statistical significance (*P* ˂ .01), the language bias in literature selection may overestimate the true clinical benefits.

#### 3.3.2. Bacterial clearance

**Figure 5.**
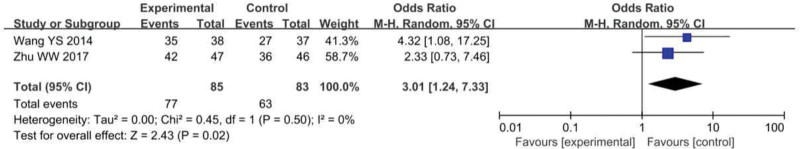
Bacterial clearance rate: Yinhua Miyanling tablet + Levofloxacin capsules versus Levofloxacin capsules.

Two studies^[8,13]^ were conducted to compare the effects of silver Yinhua Miyanling tablet + Levofloxacin capsules vs Levofloxacin capsules on bacterial clearance, with 85 patients in the experimental group and 83 patients in the control group. Heterogeneity test results showed that *P* = .50; *I*^2^ = 0%, clinical homogeneity, using the fixed effect model, the results showed that OR = 3.01, 95% CI was (1.24–7.33), *P* ˂ .05, the bacterial clearance rate was statistically significant. See Figure 5.

#### 3.3.3. Recurrence rate

**Figure 6.**
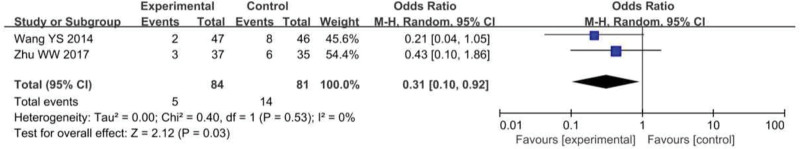
Recurrence rate: Yinhua Miyanling tablet + Levofloxacin capsules versus Levofloxacin capsules.

Two studies^[8,13]^ compared the effects of Yinhua Miyanling tablet + Levofloxacin capsule vs Levofloxacin capsule on recurrence rate, with 84 patients in the experimental group and 81 patients in the control group. Heterogeneity test results showed that *P* = .53; *I*^2^ = 0%, clinical homogeneity, fixed effect model, OR = 0.31, 95% CI (0.10–0.92), *P* ˂ .05, recurrence rate was statistically significant. See Figure 6.

### 3.4. Adverse reactions

**Figure 7.**
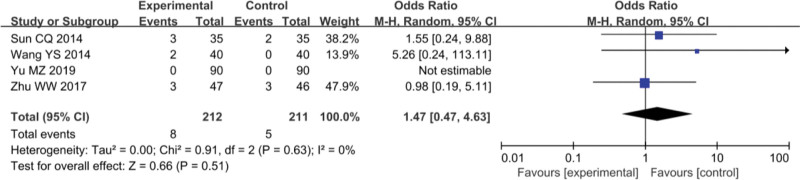
Adverse reactions, mild diarrhea: Yinhua Miyanling tablet + Levofloxacin capsules versus levofloxacin capsules.

**Figure 8.**
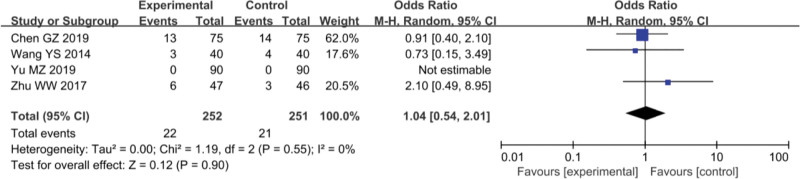
Adverse reactions, nausea, and abdominal discomfort: Yinhua Miyanling tablet + Levofloxacin capsules versus levofloxacin capsules.

In the study of Yinhua Miyanling tablet + Levofloxacin capsules vs Levofloxacin capsules, 4 studies^[8,9,11,13]^ mentioned adverse reactions of mild diarrhea, including 212 patients in the experimental group and 211 patients in the control group. Heterogeneity test results showed that *P* = .63; and *I*^2^ = 0%, showing clinical homogeneity. Using the fixed-effect model, the results showed that OR = 1.47, 95% CI was (0.47–4.63), *P* > .05, as shown in Figure 7. Adverse reactions of nausea and gastrointestinal discomfort were mentioned in 4 studies.^[8,10,13,16]^ Heterogeneity test results showed that *P *= .55, *I*^2^ = 0%, indicating clinical homogeneity. Using fixed-effect model, the results showed that OR = 1.04, 95%CI was (0.54–2.01), *P* > .05. There was no significant difference in adverse reactions between the studies. See Figure 8.

### 3.5. Bias evaluation

Because the number of literature included in the evaluation of each outcome indicator was <10, a funnel plot was not performed for bias evaluation. According to the improved Jadad scoring criteria,^[21]^ the quality of all the included literature was evaluated, and any disagreement between 2 independent researchers was resolved after discussion by a third party. The scoring criteria include generation of random sequence; randomized hiding; blind method; follow-up (withdrawal and withdrawal). The total score is 1 to 7, with 1 to 3 considered as low-quality studies and 4 to 7 considered as high-quality studies. See Table 2. According to the Jadad score, we found that the quality of the 14 kinds of literature included was not high, but most of the RCTS rated as low quality by the Jadad scale did not describe the blind method and random sequence generation method, which may affect the quality of the RCTS, but could be included in the systematic review.^[22]^ The main reason for being excluded from the Jadad scale is that the article does not describe the generation methods of blinding and random sequences. However, from a research perspective, these RCTS are relatively rigorously designed and the results are reliable. They are high-quality clinical studies and can be used for further systematic reviews.

**Table 2:** Results of methodological quality evaluation.

References	Research type	Random sequence generation	Allocation hiding	Blind method	Follow-up visit	Total points
Zhao^[7]^	RCT	1	1	0	0	2
Zhu et al^[8]^	RCT	1	1	0	1	3
Sun and Li^[9]^	RCT	1	1	0	0	2
Yu et al^[10]^	RCT	2	1	0	1	4
Zhao and Gao^[11]^	RCT	0	0	0	1	1
Liao et al^[12]^	RCT	1	1	0	1	3
Wang and Wang^[13]^	RCT	1	1	0	1	3
Li^[14]^	RCT	0	0	0	0	0
Tang et al^[15]^	RCT	1	1	0	0	2
Chen and Zhang^[16]^	RCT	1	1	0	0	2
Wang^[17]^	RCT	2	1	0	0	3
Zhang and Liu^[18]^	RCT	2	1	0	1	4
Zhang^[19]^	RCT	2	1	0	1	4
Zhong^[20]^	RCT	1	0	0	1	2

RCT = randomized controlled trial.

### 3.6. The quality of evidence assessment in GRADE framework

Because all the included studies were RCT studies, the initial quality of the evidence was “high.”

Overall, the quality evaluation of the included evidence was“moderate.” The limitations have not been underestimated, as the study population can well represent the target population. The inconsistency was not downgraded because the *I*^2^ value was generally low. Indirectness was not reduced because the populations, indicators and results of these studies were consistent with those of our research in this meta-analysis. Because our research sample size was sufficient and the CI was moderate, the imprecision was not reduced. Publication bias was reduced by one category because all the literature was in the same language. As we believe that the incidence of uncomplicated UTIs is very high, we have raised the rating of “big effect.” Due to publication bias, the quality of evidence assessment was reduced to “moderate” in all subgroups.

## 4. Discussion

Urinary tract infection belongs to the category of “gonorrhea syndrome” in Chinese medicine. Yinhua Miyanling Tablet is a Chinese patent medicine preparation widely used in China for the treatment of urinary tract infection. It was developed for the treatment of bladder damp-heat classical prescription Bazheng San. Compared with the ancient prescription Bazheng San, the reuse of honeysuckle can enhance the efficacy of clearing heat and detoxifying, removing rhubarb and adding mulberry parasitization, and enhancing the efficacy of Fuzheng and dispelling evils.^[23]^ Its main ingredients include Polygonum aviculare, Polygonum aviculare, Ambrosia ambrosa, Shiwei, Ambrosia ambrosa, Psyllium psyllium, bamboo leaves, mulberry parasite, and russet. Its functions are to clear heat detoxify and dislodge dampness and relieve drench. It is used for acute pyelonephritis and acute cystitis. A large number of studies have shown that the royal medicine Honeysuckle extract and its chemical components have a variety of pharmacological activities, mainly reflected in anti-inflammatory, antibacterial, and antiviral activities^.[24,25]^ The ethanol extract of the Yinhua Miyanling tablet can alter the ultrastructure of *Escherichia coli* by interfering with or destroying the cell wall of *Escherichia coli* or destroying the large molecular weight protein in the bacteria. Meanwhile, the in vivo and in vitro studies of animal experiments have shown that the Yinhua Miyanling tablet has obvious antibacterial endotoxin effects and has obvious antibacterial and anti-inflammatory effects.^[26,27]^ At the same time, it can also improve the immune function of the body, promote the inflammatory absorption of patients, and improve the renal blood flow, thus reducing the recurrence of disease and the incidence of renal fiber changes,^[17]^ and the incidence of adverse reactions after drug withdrawal is low.^[28]^

A total of 14 studies were included in this meta-analysis, with a total of 1773 patients. In the treatment of uncomplicated UTIs, the use of Yinhua Miyanling tablet alone or combined with levofloxacin capsule can significantly improve the clinically effective rate, and bacterial clearance rate, and reduce the recurrence rate, with good safety. Due to the inconsistency of dose and course of administration between the experimental group and the control group in each study, the study design was not explicitly blind and the assignment was hidden.

Given the methodological limitations identified, particularly in randomization and blinding, our GRADE score assessment for the quality of evidence is “moderate,” which means we have some confidence in the treatment of uncomplicated UTIs with Yinhua Miyanling tablet.

Our research still has some limitations. The GRADE approach requires researchers to have a high level of knowledge in the relevant field. Even so, it is difficult to eliminate the subjective judgment of researchers. Therefore, we try our best to reduce the subjective influence through parallel evaluation by multiple researchers.

It is still necessary to confirm whether the treatment of uncomplicated UTIs with Yinhua Miyanling tablet has more advantages than Western medicine.

There are some limitations in this study. Although the number of included literatures was relatively small, in validity studies, researchers were more willing to include data that proved the research results were positive, which led to the existence of publication bias. Most studies do not specify how random sequences are generated, double-blind and hidden operations are not used in the study design, and some studies do not carry out follow-up or intentionality analysis. There may be bias in selection, implementation, and measurement, which affects the strength of this study. To elaborate further. Among the 14 studies, only 6 mentioned the randomization method, while the rest did not. None of the studies mentioned blinding or concealment measures, and 5 studies conducted relevant follow-ups. Due to insufficient reporting on randomization, allocation concealment and blinding, there is a high degree of selection bias, which also reduces the quality of the research. Insufficient randomization such as pseudo-randomization methods may lead to an imbalance in baseline characteristics between groups and confuse the true assessment of the intervention effect. Insufficient allocation concealment such as unsealed allocation sequences may enable researchers to selectively include subjects, undermining the fairness of randomization. Insufficient blinding such as when the subjects or researchers are aware of the groupings can introduce subjective biases, such as the placebo effect or assessment bias. These flaws collectively weaken the internal validity of the study, making the observed effects likely to stem from bias rather than the actual intervention effect, and ultimately leading to unreliable research results.

The predominant inclusion of Chinese-language studies inherently introduces linguistic and regional bias, as TCM research is primarily published in journals targeting Mandarin-speaking audiences. This limitation may exclude relevant studies published in other languages that could offer divergent perspectives or methodologies. While our search strategy adhered to systematic review standards, the scarcity of non-Chinese TCM literature reflects broader academic disparities, including uneven research funding, publication preferences, and cultural barriers to international collaboration. Such bias potentially skews the applicability of findings toward specific demographic or practice patterns prevalent in Chinese clinical settings. Future reviews could mitigate this by collaborating with multilingual teams to identify gray literature or regional databases. Additionally, the observed homogeneity in results may partly stem from linguistic uniformity rather than therapeutic consensus, underscoring the need for cautious interpretation when generalizing conclusions to global contexts.

Variability in treatment regimens, dosages, and durations among included trials is a significant source of clinical and methodological heterogeneity in systematic reviews and meta-analyses. This variability can arise from differences in study design, clinical guidelines across regions, evolving treatment protocols over time, or investigator preferences. For instance, some trials may test higher or more frequent dosing to maximize efficacy, while others may use lower doses to minimize adverse effects. Transparent reporting of these differences is essential to assess the generalizability of findings and guide clinical decision-making.

In summary, Yinhua Miyanling tablet has a high clinical effective rate, a low bacterial clearance rate, and a low recurrence rate in the treatment of uncomplicated UTIs, with good safety. Limited by the methodological quality and sample size of the included studies, this recommendation requires confirmation by rigorously designed trials.

So, future RCTs should prioritize: computer-generated randomization with allocation concealment; standardized blinding procedures; and predefined outcome assessment protocols to minimize observer bias.

## Author contributions

**Data curation:** Yanyan Zhou, Ming Li, Yiwen Tang, Xiong Wang, Qi Li, Ding Li, Feng Xu, Ruoyu Qiao, Zejia Zhang, Yongqiang Wei, Pengyu Cheng, Lihong Liu, Rongfu Ji, Xiaofeng Jiang.

**Formal analysis:** Yanyan Zhou, Ming Li, Yiwen Tang, Xiong Wang, Qi Li, Ding Li, Feng Xu, Ruoyu Qiao, Zejia Zhang, Yongqiang Wei, Pengyu Cheng, Lihong Liu, Rongfu Ji.

**Investigation:** Zhan Gao.

**Writing – original draft:** Jiasen Ding.
